# Effect of temperature and water potential on the germination of seeds from three different populations of *Bidens pilosa* as a potential Cd hyperaccumulator

**DOI:** 10.1186/s12870-022-03876-3

**Published:** 2022-10-12

**Authors:** Rui Zhang, Dali Chen, Huizhuan Liu, Changlin Guo, Li Tang, Honggang Wang, Yinhua Chen, Kai Luo

**Affiliations:** 1 School of Tropical Crops, Hainan University, Haikou, 570228 China; 2 College of Pastoral Agriculture Science and Technology, Lanzhou University, Lanzhou, 730000 China

**Keywords:** Seed, Germination, Hyperaccumulator, Temperature, Water potential

## Abstract

**Background:**

*Bidens pilosa* L., an annual herb, has recently been shown to be a potential Cd-hyperaccumulating plant. The germination characteristics of *B. pilosa* have been documented, while the difference among populations remains unclear. Understanding variability in seed germination among populations is crucial for determining which populations to use for soil remediation programs.

**Results:**

Present study was conducted to compare the requirements of temperature and water potential for germination of *B. pilosa* cypselae (the central type, hereafter seeds) from three populations using the thermal time, hydrotime, and hydrothermal time models. Seeds of three populations were incubated at seven constant temperatures (8, 12, 15, 20, 25, 30, and 35 °C) and at each of four water potentials (0, -0.3, -0.6, and -0.9 MPa). The results showed that germination percentage and rate of *B. pilosa* seeds were significantly by population, temperature, water potential and their interaction except for the interaction of population and water potential. Seeds from Danzhou population displayed a higher base temperature (*T*_b_) for germination than those from Guilin and Baoshan population, however the ceiling temperature (*T*_c_) had no consistent level among the populations but varied according to the water potential. In addition, the median base water potential [*ψ*_b(50)_] for germination of seeds from Danzhou population was higher than that for seeds from Baoshan and Guilin population at low temperatures (< 25 °C), which was opposite at high temperatures (≥ 25 °C).

**Conclusion:**

Seed germination requirements of *B. pilosa* on temperature and water differed significantly among populations. Differences in seed germination among populations may be complicated, which could not be simply explained by the temperature and rainfall conditions where the seeds were produced as previously reported. The results suggested that programme management should consider variation in seed germination traits when select which population could be applied to what kind of target remediation sites.

## Background

Cadmium (Cd), produced by industrial activities such as mining, electroplating, and waste irrigation, is one of the most hazardous and ubiquitous contaminants in soil and water [1]. Thus, it remains a possible health risk to humans due to bioaccumulation in the food chain, which can lead to lethal disorders like “itai-itai disease” [2]. Therefore, cleanup of Cd-contaminated soil is increasingly imperative. The concept of phytoremediation was first introduced by Chaney et al. (1999), who recommended leveraging the accumulation capability of hyperaccumulator plants to remove heavy metals from the soil which is a cost-effective, environmentally friendly, and sustainable method [3, 4]. To date, over 400 plant species have been discovered as natural hyperaccumulators around the world, but fewer than 10 naturally occurring species have been reported to hyperaccumulate Cd, including *Amaranthus hybridus*, *Arthrocnemum macrostachyum*, *Chara aculeata*, and *Phytolacca americana* [5].

*Bidens pilosa* L. (Asteraceae), is an annual herb that grows widely from the tropics to subtropical zones. After being first reported in Hong Kong in 1857, *B. pilosa* has spread and is distributed throughout east, central south, and southwest China [6]. It's been advocated as a human treatment for diseases including protozoan infection, bacterial infection, gut disorders, immunological disorders, and so on [7]. Over 200 phytochemicals have been identified from *B. pilosa*, including polyacetylenes and flavonoids proposed as the active compounds in treating malaria [8]. Recently, it has been proven to be a potential Cd-hyperaccumulating plant [9], which has the characteristics of higher biomass, faster growth, higher seed production, and greater tolerance to adverse environmental conditions than other Cd hyperaccumulators [1].

Seed germination is the first crucial growth stage for successful seedling establishment for soil remediation programs and is affected by various environmental factors, among which temperature and moisture are of overriding importance [10–16]. It is notable that seeds from different environmental conditions of a species’ range often show variability in germination requirements [17–20]. All seeds require a specific range of temperatures for germination; this is called the cardinal temperature: the base temperature (*T*_b_), optimal temperature (*T*_o_), and ceiling temperature (*T*_c_), which varies according to the climate conditions under which the seeds originated [21–24]. Additionally, the base water potential required for seed germination varies widely among seeds from different provenances, populations, or geographic locations [20, 25–27]. It is crucial for land managers to understand the variations in germination requirements for seeds from diverse populations to ensure that the seeds are sown at the most favorable time and conditions to support germination for establishment of robust plants that can accomplish “phytoremediation” [12, 21].

To date, seed germination requirements for temperature and moisture have been well documented through utilization of the thermal time, hydrotime, and hydrothermal time models [28–30]. Research on threshold values has been mostly conducted for agricultural and ecological purposes in species, such as *Carthamus tinctorius* [29], *Brassica napus* [31], *Stipa* species [32], *Camelina sativa* [33], and *Alyssum homolocarpum* [34]. In addition, previous studies on *B. pilosa* often focused on its metabolites as an edible medicinal plant [8, 35–37], the plant’s mechanism of Cd accumulation/translocation [1, 38], the reasons for its successful invasiveness [39], and the alleviation of dormancy as a weedy species [40]. Previous studies showed that seed germination of *B. pilosa* could occur over a wide range of temperatures [41–44], and light greatly stimulated seed germination [43]. In addition, *B. pilosa* was found to germinate at high salt levels (13% at 100 mM NaCl and 3% at 200 mM NaCl), but preferred a moist environment that less than 3% of the seed germinated at -0.75 MPa and germination ceased at -0.8 MPa [41, 43]. However, collaborative response of various environmental factors such as temperature and water on seed germination of *B. pilosa* had not been studied. In particular, differences in seed germination characteristics among populations have been reported by many previous studies [24, 26, 31], but little research on this species [45, 46].

Cd pollution mainly happened in the southwest, central and north China including Yunnan, Guangxi, Guangdong and other areas where there were rich in cadmium resource [47], meanwhile combined the widely distribution of *B. pilosa* in China [48, 49]. Thus, we chose seeds collected from Baoshan population in Yunnan Province, Guilin population in Guangxi Province and Danzhou population in Hainan Province to test the effect of temperature and water potential on the seed germination of *B. pilosa*. Further used the thermal time, hydrotime, and hydrothermal time models to compare seed germination requirements among populations based on the experimental data. The results of this study will be useful for understanding variability in the seed germination requirements of *B. pilosa* across a range of populations, which will lead to improved seed sourcing and seed application timing decisions for soil remediation programs.

## Results

### Effect of temperature and water potential on the percentage and rate of *B. pilosa* seed germination

Population, temperature, water potential, and all their interactions had significant effects on the seed germination percentage and rate of *B. pilosa,* except for the interaction between population and water potential (Table 1). With increasing temperature, the final germination percentage and germination rate climbed and then fell at all water potentials, while they declined as the water potential fell at all temperatures for the three populations (Table 2). The highest germination percentage and germination rate were observed at 25 °C for the three populations, suggesting that 25 °C was likely considered the optimal temperature for seed germination of *B. pilosa*. From the results, we found that seed germination percentage at 8 °C under -0.6 MPa was shown as Baoshan > Guilin > Danzhou, while was shown as Danzhou > Guilin > Baoshan at 35 °C under -0.6 MPa. Then the thermal time, hydrotime, and hydrothermal time models were utilized to investigate the seed germination response to temperature and water potential further. Based on the seed germination rate (1/t_50_), 8, 10, 15, and 20 °C were determined as the suboptimal temperatures and 25, 30, and 35 °C as the supraoptimal temperatures. The meanings of all the parameters in present study were shown in Table 3.

**Table 1:** Effect of population, temperature and water potential on seed germination percentage and rate (1/t_50_) of *B. pilosa* were analyzed by GLMMs based on binomial distribution

Source of variation	Chi	Germination percentage (%)		Chi	Germination rate (1/t_50_)	
		*df*	*P*		*df*	*P*
Population (*P*)	40.618	2	0.000***	9.816	2	0.007**
Temperature (*T*)	69.607	6	0.000***	1047.690	6	0.000***
Water potential (*W*)	95.603	3	0.000***	7.620	2	0.022*
*P* × *T*	36.225	12	0.000***	88.658	12	0.000***
*P* × *W*	2.242	6	0.896	5.980	4	0.200
*T* × *W*	36.106	18	0.007**	422.804	12	0.000***
*P* × *T* × *W*	63.701	36	0.003**	104.628	24	0.000***

^*^P < 0.05

^**^P < 0.01

^***^P < 0.001

**Table 2:** The effect of temperature and water potential on seed germination percentage and rate of *B. pilosa* from three populations

Population	Temperature (℃)	Germination percentage (%)				Germination rate (1/t_50_)			
		0 MPa	-0.3 MPa	-0.6 MPa	-0.9 MPa	0 MPa	-0.3 MPa	-0.6 MPa	-0.9 MPa
Baoshan	8	80.00c	74.67b	26.00c	0.00a	0.05d	0.05e	0.00c	-
	12	94.00ab	96.00a	82.67a	4.00a	0.15c	0.13c	0.07b	-
	15	98.67a	96.00a	92.00a	2.67a	0.19c	0.14c	0.09a	-
	20	98.00ab	93.33a	88.67a	3.33a	0.32b	0.18a	0.09a	-
	25	97.33ab	95.33a	86.00a	8.00a	0.43a	0.16b	0.07b	-
	30	92.00ab	86.00ab	38.00b	0.00a	0.37b	0.08d	0.00c	-
	35	89.33c	41.33c	4.67d	0.00a	0.13c	0.00f	0.00c	-
Guilin	8	76.67b	68.67b	20.67c	0.00b	0.05e	0.04e	0.00d	-
	12	96.67a	94.00a	69.33b	3.33b	0.10d	0.12d	0.07b	-
	15	98.67a	98.00a	85.33ab	3.33b	0.15d	0.15 cd	0.07b	-
	20	96.00a	94.00a	94.67a	6.00b	0.41b	0.21b	0.11a	-
	25	98.67a	99.33a	84.67ab	16.00a	0.50a	0.27a	0.08b	-
	30	97.33a	94.00a	65.33b	4.67b	0.32c	0.17bc	0.04c	-
	35	82.67b	69.33b	23.33c	0.00b	0.10d	0.05e	0.00d	-
Danzhou	8	48.00d	30.00d	7.33d	0.00c	0.00e	0.00d	0.00c	-
	12	95.33ab	96.00ab	93.33a	9.33b	0.13d	0.09c	0.07b	-
	15	94.00ab	96.00ab	82.00ab	8.67b	0.16d	0.10bc	0.06b	-
	20	97.33a	100.00a	92.67a	12.67ab	0.37b	0.29a	0.10a	-
	25	97.33a	95.33ab	88.00a	16.00a	0.53a	0.29a	0.10a	-
	30	86.67c	86.67b	74.00b	8.00b	0.31c	0.14b	0.10a	-
	35	88.67bc	59.33c	31.33c	0.67c	0.15d	0.04d	0.00c	-

Different lowercase letters indicate significant differences among different temperatures at the same water potential within each population (DUNCAN, *P* < 0.05). “-” means no data have been calculated

**Table 3:** The meanings of the parameters in present study

Parameter	Description
*T*_b_	the base temperature
*T*_o_	the optimal temperature
*T*_c_	the ceiling temperature
*t*_g_	the time to a given specific germination percentage g
*θ*_T(50)_	the thermal time for 50% of seeds to germinate
*T*_c(50)_	the ceiling temperature for 50% of seeds to germinate
*θ*_T_	the thermal time constant
*σ*_θT_	the standard deviation of log *θ*_T_ requirements
*σ*_Tc_	the standard deviation of *T*_c_
*θ*_H_	the hydrotime constant
*ψ*_b(50)_	the median base water potential
*σ*_ψb_	the standard deviation of *ψ*_b(50)_
*θ*_HT_	the hydrothermal time constant

### Thermal time model

**Fig. 1:**
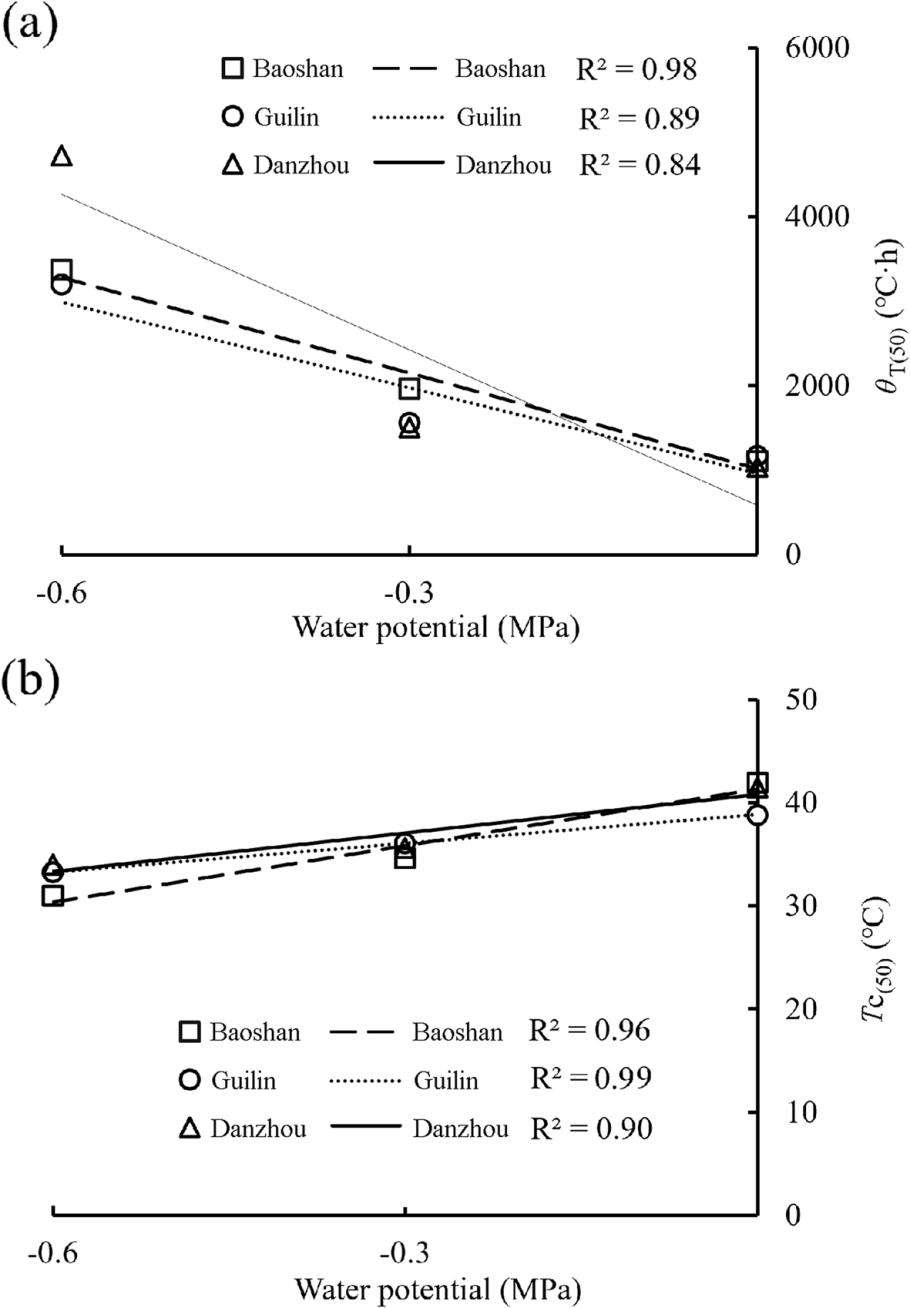
The relationship between the temperature threshold and water potential. **a** the thermal time [*θ*_T(50)_] at suboptimal temperatures,** b** the ceiling temperature [*T*_c(50)_] at supraoptimal temperatures

The estimated values of the base temperature (*T*_b_), the thermal time for 50% of seeds to germinate [*θ*_T(50)_], the ceiling temperature for 50% of seeds to germinate [*T*_c(50)_] and the thermal time constant (*θ*_T_) were varied with water potentials. The *T*_b_ at 0 and -0.3 MPa was shown to be Danzhou > Guilin > Baoshan. The order of the *T*_c_ was Baoshan > Danzhou > Guilin at 0 MPa, Guilin > Danzhou > Baoshan at -0.3 MPa, and Danzhou > Guilin > Baoshan at -0.6 MPa (Table 4). A decreased *T*_b_ and *T*_c_ associated with water stress would limit the temperature range of germination under water stress conditions, especially for Baoshan population. The estimated values of the *θ*_T(50)_ and the *T*_c(50)_ is plotted against water potential, and the *θ*_T(50)_ increased as the water potential decreased at suboptimal temperatures (Fig. 1a), and the *T*_c_ decreased as the water potential decreased at supraoptimal temperatures for all the three populations (Fig. 1b).

**Table 4:** Seed germination parameters of *B.* *pilosa* from three populations based on thermal-time model analysis at suboptimal and supraoptimal temperature for different water potentials

Population	Water potential (MPa)	suboptimal temperature				supraoptimal temperature			
		*θ*_T(50)_ (℃·h)	*σ*_θT_	*T*_b_(℃)	*R*^2^	*θ*_T_ (℃·h)	*σ*_Tc_	*T*_c(50)_ (℃)	*R*^2^
Baoshan	0	1111.92	0.65	5.38	0.92	890.68	7.30	41.96	0.82
	-0.3	1969.45	0.66	4.58	0.91	1326.15	3.75	34.63	0.93
	-0.6	3374.73	0.69	4.41	0.84	1919.41	3.24	30.97	0.95
Guilin	0	1173.11	0.76	5.95	0.80	637.28	5.30	38.84	0.87
	-0.3	1561.64	0.67	5.58	0.87	825.91	5.94	36.07	0.72
	-0.6	3201.87	0.63	5.10	0.90	2195.41	6.29	33.25	0.89
Danzhou	0	1039.63	0.77	6.81	0.91	823.21	6.58	41.52	0.87
	-0.3	1505.51	0.68	6.66	0.89	733.08	6.45	35.64	0.85
	-0.6	4725.14	0.59	5.14	0.76	1596.29	6.14	34.04	0.81

*θ*_T(50)_ = thermal time for 50% of seeds to germinate, *σ*_θT_ = standard deviation of log *θ*_T(50)_, *T*_b_ = constant base temperature in suboptimal temperature range. *θ*_T_ = constant thermal time, *σ*_Tc_ = standard deviation for *T*_c(50)_ at supraoptimal temperature, *T*_c(50)_ = maximum temperature for 50% of seeds to germinate

### Hydrotime model

**Fig. 2:**
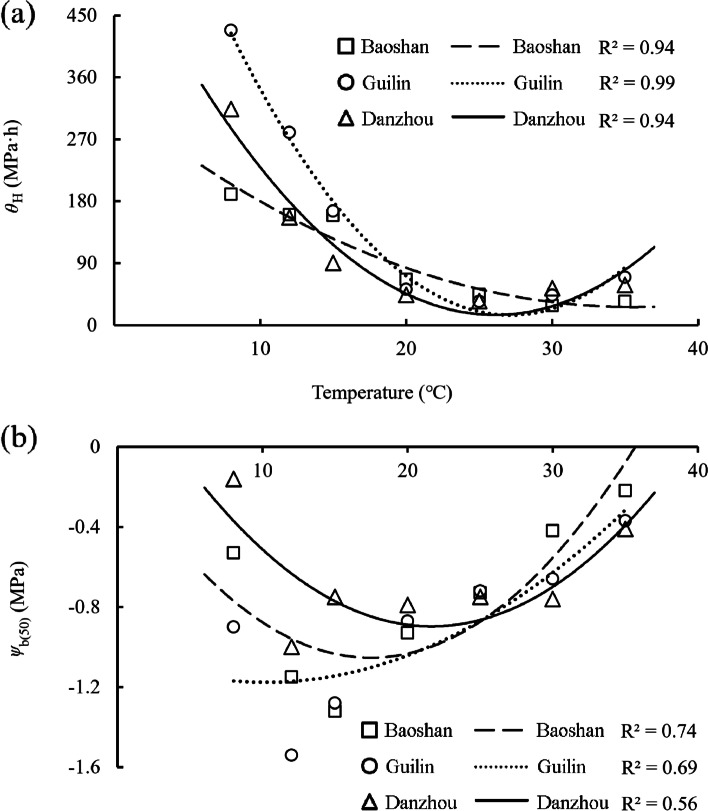
The relationship between the water potential threshold and temperature. **a** the hydrotime constant (*θ*_H_),** b** the median base water potential [*ψ*_b(50)_]

The hydrotime time models were fitted to seed germination data of *B. pilosa* under different temperature regimes, and the estimated values are shown in Table 5. The hydrotime constant (*θ*_H_) decreased and then increased with increasing temperature for all three populations, and the lowest value was obtained at 30 °C for Baoshan population, and at 25 °C for Guilin and Danzhou population. The median base water potential [*ψ*_b(50)_] decreased and then increased with increasing temperature for all three populations, and the lowest value was obtained at 15 °C for Baoshan population, and at 12 °C for Guilin and Danzhou population. The estimated values of the *ψ*_b(50)_ and the *θ*_H_ is plotted against temperature, and which decreased and then increased with increasing temperature for all the three populations (Fig. 2).

**Table 5:** Seed germination parameters for response of *B. pilosa* to water potential from three populations based on hydrotime model analysis for different temperatures

Population	Temperature (℃)	*θ*_H_ (MPa·h)	*ψ*_b(50)_ (MPa)	*σ*_ψb_	*R*^2^
Baoshan	8	190.20	-0.53	0.56	0.73
	12	160.23	-1.15	0.48	0.85
	15	159.07	-1.32	0.52	0.89
	20	66.06	-0.93	0.37	0.77
	25	44.09	-0.73	0.27	0.64
	30	28.06	-0.42	0.25	0.78
	35	34.01	-0.22	0.16	0.93
Guilin	8	428.40	-0.90	0.43	0.86
	12	279.78	-1.54	0.82	0.74
	15	165.44	-1.28	0.66	0.81
	20	52.00	-0.87	0.31	0.74
	25	33.96	-0.72	0.26	0.77
	30	43.23	-0.66	0.29	0.80
	35	69.56	-0.37	0.36	0.79
Danzhou	8	313.91	-0.16	0.68	0.81
	12	156.35	-1.00	0.47	0.64
	15	90.21	-0.75	0.36	0.75
	20	43.81	-0.79	0.30	0.73
	25	34.79	-0.75	0.24	0.83
	30	53.34	-0.76	0.47	0.77
	35	58.23	-0.41	0.30	0.93

*θ*_H_ = constant hydrotime, *ψ*_b(50)_ = median base water potential, *σ*_ψb_ = standard deviation of *ψ*_b(50)_

Compared with Baoshan and Guilin populations, tolerance to water potential for seeds from Danzhou population was more sensitive to temperature. Our analyses revealed that the *ψ*_b(50)_ was essentially equal for the three populations at 25 °C, while seeds from Danzhou population were more tolerant to water stress at high temperature (T ≥ 25 °C), and those from Guilin and Baoshan population were more tolerant to water stress at lower temperatures (T < 25 °C) (Table 5).

### Hydrothermal time model

**Fig. 3:**
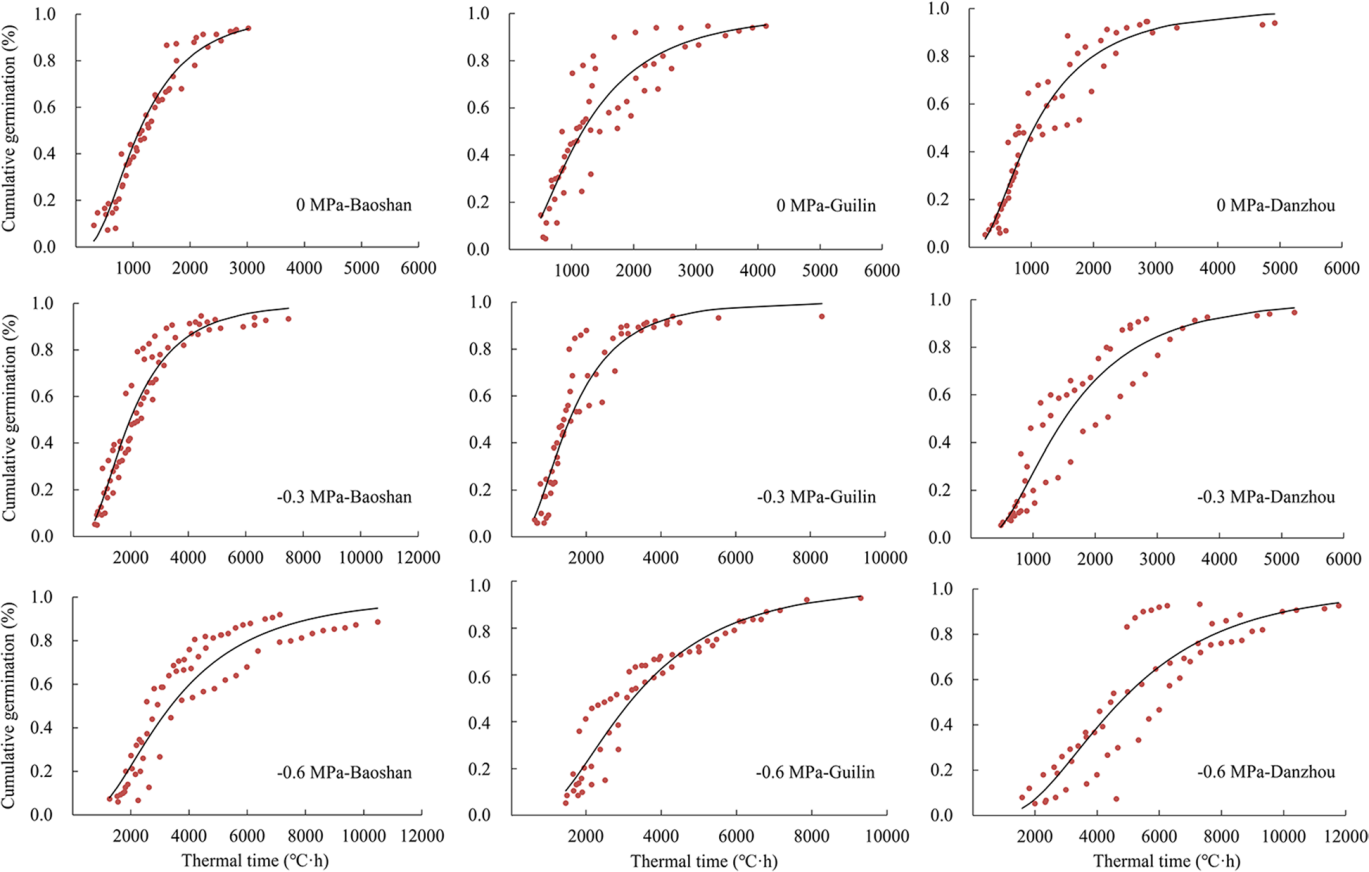
Thermal time of *B. pilosa* seeds from three populations predicted by thermal time models based on Normal distribution at different water potentials at suboptimal temperatures. The red circles show the observed mean thermal times and the black dashed lines show the predicted thermal times fitted by the thermal time model

**Fig. 4:**
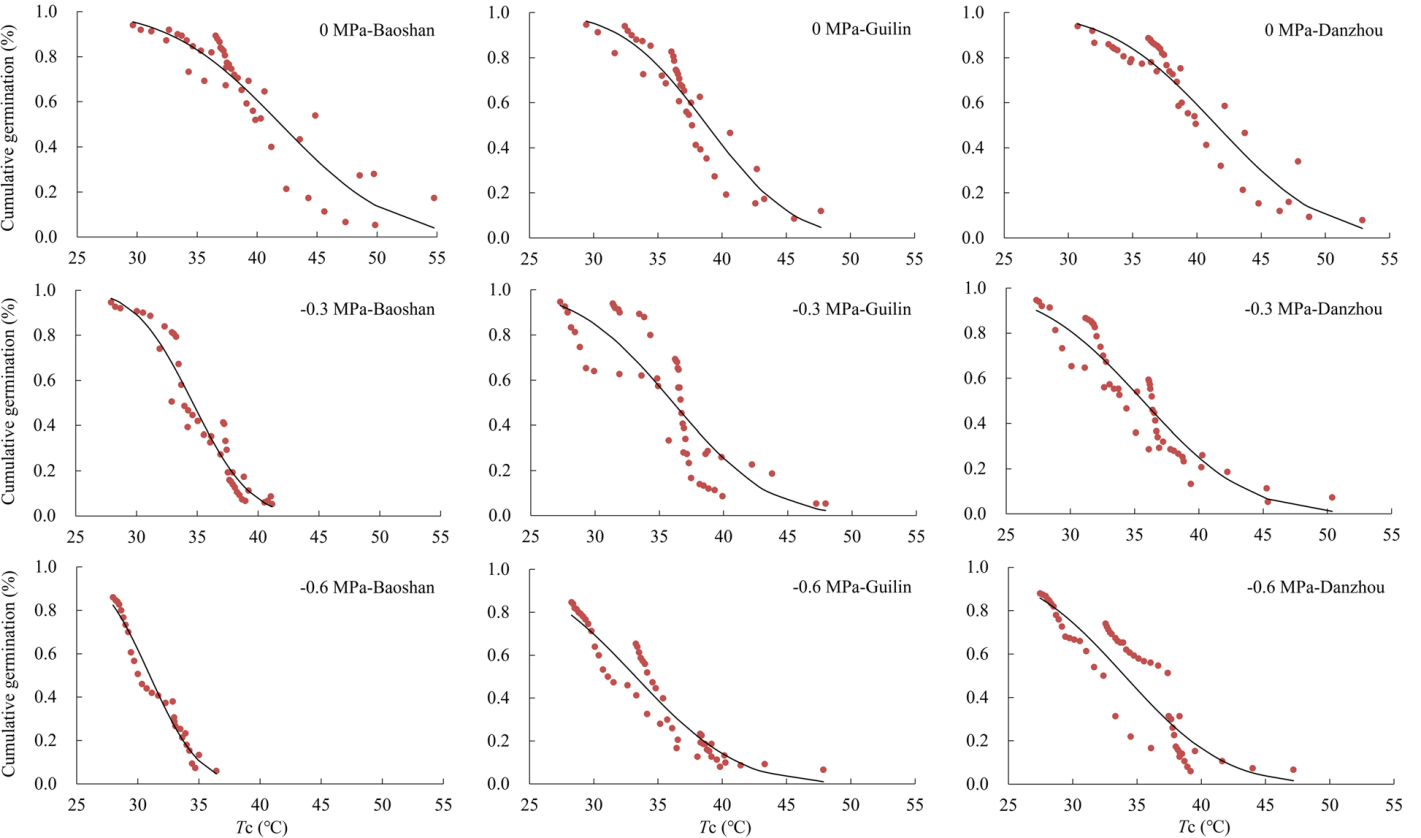
Ceiling temperature of *B. pilosa* seeds from three populations predicted by thermal time models based on Normal distribution at different water potentials at supraoptimal temperatures. The red circles show the observed mean ceiling temperature and the black dashed lines show the predicted the ceiling temperature fitted by thermal time model

The hydrothermal time model parameters were utilized to explain the difference in germination timing of different populations over the combined range of temperatures and water potentials at which germination can occur. Based on the hydrothermal time model parameters displayed in Table 6, the estimated hydrothermal time constant (*θ*_HT_) and *ψ*_b(50)_ are shown as Baoshan > Guilin > Danzhou, while the estimated *T*_b_, optimal temperature (*T*_o_) and *θ*_H_ values were opposite; that is, Danzhou > Guilin > Baoshan. The value of *θ*_HT_ indicated that the hydrothermal time required for completing germination for seeds from Danzhou population was shorter than those from Guilin and Baoshan population, and differences in *ψ*_b(50)_ values demonstrated that seeds from the Danzhou population were more sensitive to low water potential than those from the Guilin and Baoshan populations. The fits between seed germination and the thermal time at different water potentials at suboptimal temperatures (Fig. 3) and the fits between seed germination and the ceiling temperature at different water potentials at supraoptimal temperatures (Fig. 4) showed well agreements between the predicted and observed values for the Normal distribution.

**Table 6:** Seed germination parameters for response of *B. pilosa* to temperature and water potential from three populations based on hydrothermal time model analysis

Population	suboptimal temperature					supraoptimal temperature					
	*θ*_HT_ (MPa·°C·h)	*T*_b_ (°C)	*ψ*_b(50)_ (MPa)	*σ*_ψb_	*R*^*2*^	*k*_T_	*T*_o_ (°C)	*θ*_H_ (MPa·h)	*ψ*_b(50)_ (MPa)	*σ*_ψb_	*R*^2^
Baoshan	1303.52	4.33	-1.12	0.57	0.71	0.05	15.53	36.72	-1.12	0.24	0.73
Guilin	1095.89	5.96	-1.09	0.53	0.76	0.05	18.96	40.39	-1.09	0.30	0.72
Danzhou	694.30	6.47	-0.79	0.39	0.69	0.05	25.72	40.94	-0.79	0.32	0.75

## Discussion

Knowledge of variation in seed germination requirements among populations helps to plan effective strategies for seed selection and to understand where a species can grow and when to sow in soil remediation programs [12, 50]. Our preliminary results together with previous studies indicated that the thermal time, hydrotime, and hydrothermal time models could help to quantify seed germination behavior against various temperatures and water potentials [11, 29, 33, 50–53]. Seed germination percentage and rate of *B. pilosa* declined with decreasing water potential while increased and then decreased with increasing temperature for the three populations. Seed germination patterns of *B. pilosa* response to temperature and water potential varied among populations. Furthermore, seed germination requirements on temperature were affected by water potential and germination requirements on water potential were also influenced by temperature. Differences in seed germination behavior among populations within the range of this species could be particularly suited to increase their fitness under global climate change.

Seeds from different populations may differ greatly in their base, optimal, and ceiling germination temperatures [23, 54–56]. Seeds from Baoshan populations showed a lower *T*_b_ than those collected from Guilin and Danzhou population. Consistent with previous studies in *Campanula americana* [57], *Cakile edentula* [20], and *Conyza bonariensis* [24], which showed that seeds from cool environments had the ability to germinate at cold temperatures. This pattern suggested that seeds with low *T*_b_ could accumulate more heat units in a given time and would germinate faster than those from warm conditions, which would maximize the length of the growing season to ensure their growth and reproduction in cold regions [58]. As proposed by Cochrane et al. (2014), there had significant positively correlation between *T*_o_ and mean annual temperature at seed source for four congeneric *Banksia* species [59]. The order of *T*_o_ for seed germination of *B. pilosa* was Baoshan < Guilin < Danzhou in present study (Table 6), perhaps related to the annual mean temperature where the mother plant grew, but still need much more evidence to confirm. However, Barros et al. (2017) found that the regardless of seed origin, a temperature of 15 °C resulted in maximum germination of *B. pilosa* in the shortest time, and that some places of origin dormant seeds can partially explain the reason [60].

In addition to temperature, water potential is another important component in regulating seed germination and seedling growth in different plant species [33, 61, 62]. The results of this study demonstrated that seed germination response to water stress varied among different populations. Germination of seeds from Danzhou population had lower *ψ*_b(50)_ at high temperatures, whereas seeds from Guilin and Baoshan had lower *ψ*_b(50)_ at low temperatures. In general, seeds from dry habitats are more tolerant to water stress than those from habitats with wet conditions [12, 26]. For example, the highest osmotic tolerance of *Silybum marianum* was obtained from a location with a dry climate and the lowest mean annual precipitation [63]. However, seed tolerance to water stress is the confluence of several environmental conditions but not just a single factor. Seeds of *Cakile edentula* from the temperate climate zone displayed higher base water potential than those from the subtropical area because the high temperature increased evaporation and sand in the subtropical area could not hold sufficient water [20]. These results indicated that differences among populations in response to water potential could not be explained simply due to rainfall where seeds produce, which likely interacts with other factors, evaporation, soil moisture level, and temperature [13, 64, 65].

We identified an interaction effect between water potential and temperature in *B. pilosa*. The *T*_b_ for seed germination decreased as the water potential decreased at suboptimal temperatures (Table 4), similar to red fescue [61] and *Camelina sativa* [33], but inversely with other studies in *Allium cepa* and *Daucus carota* [66], *Hordeum spontaneum* and *Phalaris minor* [62]. The *θ*_T(50)_ for seed germination of *B. pilosa* increased with decreasing water potential for all the three populations (Table 4), indicating that seeds required greater thermal time to complete germination due to the slow germination rate under low water potential [29, 67]. The estimated ceiling temperature decreased linearly with decreasing water potential (Fig. 1b) at supraoptimal temperatures, as seem in other plants [14, 62]. This phenomenon means that seeds will not germinate when exposed to high temperature, especially at lower water potentials, which would ensure germination occurred in suitable conditions [11]. Such interacting effects could appear in different ways that *ψ*_b(50)_ could change considerably with temperature [68–70], which increased with temperature in Kentucky bluegrass (*Poa pratensis*) and red fescue (*Festuca rubra* ssp. *litoralis*) but decreased in perennial ryegrass (*Lolium perenne*) [61]. The base water potential of *Carthamus tinctorius* decreased with increasing temperature from 5 to 20 °C but increased from 20 to 40 °C [29], which had the same trends as *B. pilosa,* but the turning point varied with population (Fig. 2b). This indicates that seeds are able to germinate with an elevated level of water stress under suitable temperature conditions [52]. It could be viewed as an adaptive strategy that reduces the accumulated hydrotime necessary for germination while increasing the probability of seed survival and seedling establishment under unfavorable conditions.

Seeds from Danzhou population showed lower *θ*_HT_, higher *ψ*_b(50)_ and higher *T*_b_ than those from Guilin and Baoshan populations (Table 6). It suggested that seeds from Danzhou population tend to germinate more rapidly in the absence of water stress and low temperature restriction, but they are severely inhibited at lower water potential and temperature. For instance, germination rate for seeds from Danzhou population was higher under 0 and -0.3 MPa at 20 and 25 °C than those from the Guilin and Baoshan populations, whereas germination was strongly inhibited under 0 and -0.3 MPa at 8 °C compared with seeds from Guilin and Baoshan populations (Table 2). This result suggested that except for the hydrothermal time model, separate models at sub- and supra-optimal temperatures should be used in modeling germination, in agreement with previous studies [11, 66]. Seeds from the Baoshan population have a lower *ψ*_b(50)_ and a larger standard deviation (*σ*_ψb_) than those from Guilin and Danzhou populations (Table 6). In accordance with research on three dry land species (*Danthonia caespitosa*, *Atriplex nummularia* and *A. vesicaria*), seeds with lower *ψ*_b(50)_ and larger *σ*_ψb_ could result in spreading germination across multiple rain events in a given year because of a higher germination plasticity [71].

## Conclusions

The findings of this study demonstrated that seed germination requirements on temperature and water of *B. pilosa* varied among populations. The estimated *T*_b_ and *ψ*_b(50)_ varied with water potential and temperature, respectively. Based on hydrothermal time model analysis, we found that seeds from Baoshan population were more tolerance to low temperature and low water potential than those from Guilin and Danzhou population. Although this information provide some suggestion on selecting seeds for soil remediation programs, further investigation is necessary under nature field environments to verify these findings. Whilst *B. pilosa* are plants with strong stress and disturbance resistances and such could be very useful as a potential Cd hyperaccumulator in phytoremediation technology theory and practice in many studies. It is worth noting that this species has a certain invasiveness, thus the plants should be mowed at or before the flowering period in practical applications.

## Materials and methods

### Seed sources

Cypselae of *B. pilosa* var. *radiata* (identification based on Chen et al. (2021) [49]) were collected from three populations located in Baoshan in Yunnan Province, Guilin in Guangxi Province, and Danzhou in Hainan Province in June 2021. The cypselae of each population were collected from several hundred plants, cleaned by hand in the laboratory, and then stored dry at 4 °C in a paper bag until use. There have two cypselae types (the peripheral type shorter and the central type longer) and the central type (more numerous than the shorter, hereafter seeds) were selected to conduct the germination experiments within two weeks of harvest. The climate and detailed information related to the three seed populations are shown in Table 7. The climate data are from weather stations near the seed collection sites, which were obtained from the Yunnan, Guangxi and Hainan Meteorological Service, respectively.

**Table 7:** Information of seed collection location, climate information, seed morphology, thousand seed weight and initial germination percentage of *B. pilosa* from three populations

Population	Longitude (E)	Latitude (N)	Altitude (m a.s.l)	Monthly mean high temp. (°C)			Monthly mean low temp. (°C)			Monthly rainfall (mm)			Annual mean temp (°C)	Annual mean rainfall (mm)	Seed length (mm)	Seed width (mm)	Awn length (mm)	1000-seed weight (g)	Initial germination (%)
				4	5	6	4	5	6	4	5	6							
Baoshan	98°52′37″	24°58′32″	734	24	26	26	13	15	19	49.2	90.3	126.7	21.3	850	8.24 ± 0.28b	0.76 ± 0.01a	2.31 ± 0.06b	1.11 ± 0.01a	98.7
Guilin	110°18′10″	25°04′10″	150	20	28	31	16	21	25	218.3	326.7	396.5	19.1	1750	9.20 ± 0.30a	0.66 ± 0.01b	2.69 ± 0.08a	0.92 ± 0.08b	99.3
Danzhou	109°29′02″	19°30′26″	137	30	35	34	22	24	25	77.5	229.3	194.4	23.2	1816	8.60 ± 0.29ab	0.70 ± 0.02b	2.58 ± 0.09a	1.18 ± 0.01a	100.0

### Experimental designs

Four replicates of 50 seeds were placed in 9-cm-diameter Petri dishes on two sheets of filter paper moistened with 6 ml of distilled water (control) or different PEG solutions. Seeds were incubated in light (12 h/12 h, daily photoperiod) at 8, 12, 15, 20, 25, 30, and 35 °C under different water potentials of 0, -0.3, -0.6, and -0.9 MPa. The light source was white, fluorescent tubes, and the photon irradiance at the seed level was 60 μmol m^−2^ s^−1^ (400–700 nm). Water potential of PEG 6000 was determined using a Dew Point Microvolt meter HR-33 T (Wescor, Logan, Utah, USA) at different temperatures. To maintain a generally consistent water potential, Petri dishes were sealed with parafilm and seeds were transferred to new filter paper with fresh solutions every 48 h. Germination (radicle protrusion) was monitored daily for 28 days. The experimental design was the same for the three populations.

Germination rate was represented by 1/t_50_ and t_50_ is defined as the time to reach germination of 50%. The time taken for cumulative germination (t_50_) to reach 50% was estimated using a GERMINATOR package by using the visual basic module from the Microsoft Excel [72].

### Data analysis

A repeated probit regression analysis was used in the thermal time, hydrotime, and hydrothermal time models to analyze the experimental data (the models and parameters are thoroughly explained in Bradford [73]).

A thermal time model was fitted to quantify the germination data with *T* at each *ψ,* and the models are shown below at the suboptimal temperature range (Eq. 1) and at the supraoptimal temperature range (Eq. 2):

1 $$\mathrm{probit}\;(\mathrm g)=\lbrack\ln(T\mathit-T_{\mathrm b})\cdot t_{\mathrm g}\mathit-\ln(\theta_{\mathrm T(50)})\rbrack/\sigma_{\theta T}$$

2 $$\mathrm{probit}\;(\mathrm g)\hspace{0.17em}=\hspace{0.17em}\lbrack T+(\theta_{\mathrm T}\mathit/t_{\mathrm g})-T_{\mathrm c(50)}\rbrack/\sigma_{\mathrm{Tc}}$$

where *T*, *T*_b_, *t*_g_, *θ*_T(50)_, *θ*_T_ and *T*_c(50)_ are the real temperature (°C), the base temperature (°C), the time to a given specific germination percentage g (h), the thermal time for 50% of seeds to germinate at suboptimal temperatures (°C·h), the thermal time constant at supraoptimal temperatures (°C·h) and the ceiling temperature (°C) (varied among different seed percentages g in the population), respectively. *σ*_θT_ is the standard deviation of log *θ*_T_ requirements among individual seeds in the population, and *σ*_Tc_ is the standard deviation of the ceiling temperature among individual seeds in the population. A probit analysis was conducted separately for each germination water potential.

A hydrotime model was fitted to quantify the germination data with *ψ* at each T, and the model is shown below:

3 $$\mathrm{probit}\;(\mathrm g)\hspace{0.17em}=\hspace{0.17em}\lbrack\psi-(\theta_{\mathrm H}/t_{\mathrm g})-\psi_{\mathrm b(50)}\rbrack/\sigma_{\mathrm\psi\mathrm b}$$

where *θ*_H_ is the hydrotime constant (MPa·h), *ψ* is the actual water potential of the seedbed, *ψ*_b(50)_ is the median base water potential, *t*_g_ is the actual time to germination of fraction g, and *σ*_*ψ*b_ is the standard deviation in base water potential among seeds within the population. Each germination temperature was subjected to a separate probit analysis. A hydrothermal time model was fitted to explain the germination data concurrently with *ψ* and *T*, and the models are shown below at the suboptimal temperature range (Eq. 4) and at the supraoptimal temperature range (Eq. 5):

4 $$\mathrm{probit}\;(\mathrm g)\hspace{0.17em}=\hspace{0.17em}\{\lbrack(\psi-\theta_{\mathrm{HT}}/((T\mathit-T_{\mathrm b})\cdot t_{\mathrm g})\rbrack{-\psi}_{\mathrm b(50)}\}/\sigma_{\psi\mathrm b}$$

5 $$\mathrm{probit}\;(g)=\{\lbrack\psi\mathit-k_{\mathrm T}\mathit\cdot(T\mathit-T_o)\mathit-\theta_{\mathrm{HT}}\mathit/((T\mathit-T_{\mathrm b})\cdot t_{\mathrm g})\rbrack-\psi_{\mathrm b(50)}\}/\sigma_{\mathrm{\psi b}}$$

where [*k*_T_ · (*T—T*_o_)] applies only when *T* > *T*_o_ and in the supra-optimal temperature range *T—T*_b_ is equal to *T—T*_o_. *θ*_HT_ is the hydrothermal time constant (MPa·°C·h), *ψ* is the real water potential (MPa), *ψ*_b(50)_ is the base value of *ψ* inhibiting germination of 50% (MPa), *t*_g_ is the real time to germination of percentage g (h) and *k*_T_ is the line slope between *ψ*_b(50)_ and *T*_o_ < *T,* which is a constant value (MPa·°C^−1^).

All statistical analyses were conducted by SPSS 25.0 (SPSS Inc., Chicago, Illinois, USA). Generalized linear mixed models (GLMMs) were used investigate the effects of population, temperature, and water potential on the seed germination percentage and rate (1/t_50_) of *B. pilosa* by using the *glmer* function in the R package *lme4*. Population and water stress were used as fixed effects, while replicates were included as random effects in each model. Duncan's multiple range test was performed to compare the means of the seed germination percentage and rate under different temperatures at the same water potential level when significant variations were discovered. Differences between the virtual *θ*_T_ and predicted *θ*_T_ at suboptimal temperatures and differences between the virtual *T*_c_ and predicted *T*_c_ at supraoptimal temperatures were estimated based on thermal time models. Second-order polynomials were used to fit the relationship between the hydrotime constant/the base water potential and temperature.

## Data Availability

The datasets used and/or analyzed during the current study are available from the corresponding author on reasonable request.
